# The Scrapie Prevalence in a Goat Herd Is Underestimated by Using a Rapid Diagnostic Test

**DOI:** 10.3389/fbioe.2020.00164

**Published:** 2020-03-12

**Authors:** Timm Konold, John Spiropoulos, Jemma Thorne, Laura Phelan, Louise Fothergill, Brenda Rajanayagam, Tobias Floyd, Beatriz Vidana, Judith Charnley, Nadya Coates, Marion Simmons

**Affiliations:** ^1^Pathology Department, Animal and Plant Health Agency Weybridge, Addlestone, United Kingdom; ^2^Central Sequencing Unit, Animal and Plant Health Agency Weybridge, Addlestone, United Kingdom; ^3^Department of Epidemiological Sciences, Animal and Plant Health Agency Weybridge, Addlestone, United Kingdom; ^4^Animal and Plant Health England Field Delivery, Skipton, United Kingdom; ^5^TSE/BVDV Testing Laboratory, Eurofins Forensic Services, Risley, United Kingdom

**Keywords:** transmissible spongiform encephalopathy, prion, classical scrapie, goat, clinical diagnosis, immunohistochemistry, ELISA

## Abstract

Current European surveillance regulations for scrapie, a naturally occurring transmissible spongiform encephalopathy (TSE) or prion disease in sheep and goats, require testing of fallen stock or healthy slaughter animals, and outline measures in the case of confirmation of disease. An outbreak of classical scrapie in a herd with 2500 goats led to the culling of the whole herd, providing the opportunity to examine a subset of goats, take samples, and examine them for the presence of disease-associated prion protein (PrP^Sc^) to provide further information on scrapie test sensitivity, pathology, and association with prion protein genotype. Goats were examined clinically prior to cull, and the brains examined post mortem by Bio-Rad ELISA, a rapid screening test used for active surveillance in sheep and goats, and two confirmatory tests, Western blot and immunohistochemistry. Furthermore, up to 10 lymphoid tissues were examined by immunohistochemistry. Of 151 goats examined, three (2.0%) tested positive for scrapie by ELISA on brain, confirmed by confirmatory tests, and a further five (3.3%) were negative by ELISA but positive by at least one of the confirmatory tests. Only two of these, both positive by ELISA, displayed evident signs of scrapie. In addition, 10 (6.6%) goats, which also included two clinical suspects, were negative on brain examination but had detectable PrP^Sc^ in lymphoid tissue. PrP^Sc^ was detected most frequently in the medial retropharyngeal lymph node (LN; 94.4% of all 18 cases) and palatine tonsil (88.9%). Abnormal behavior and circling or loss of balance when blindfolded were the best clinical discriminators for scrapie status. None of the goats that carried a single allele in the prion protein gene associated with increased resistance to scrapie (Q_211_, K_222_, S_146_) were scrapie-positive, and the percentage of goats with these alleles was greater than expected from previous surveys. Significantly more goats that were scrapie-positive were isoleucine homozygous at codon 142 (II_142_). The results indicate that the sensitivity of the applied screening test is poor in goats compared to the confirmatory tests as gold standard, particularly for asymptomatic animals. Sensitivity of surveillance could be improved by testing retropharyngeal LN or palatine tonsil in addition to brain.

## Introduction

Scrapie is a naturally occurring transmissible spongiform encephalopathy (TSE) in sheep and goats, which causes neurological signs and ultimately leads to death. The disease is caused by misfolding of the prion protein, which makes the physiological, cellular form (PrP^C^) resistant to enzymatic digestion with proteinases (Prusiner, 1995). In the absence of a reliable and quick test in a live animal, disease suspicion is currently based on clinical examination, which needs to be confirmed in the dead animal by examination of the brain. The majority of diagnostic tests are based on the detection of the misfolded scrapie prion protein (PrP^Sc^) (Gavier-Widén et al., 2005). Two scrapie types exist, atypical and classical scrapie, which are epidemiologically distinct and produce different disease phenotypes. Contrary to classical scrapie, atypical scrapie usually affects single and older animals, the agent is less resistant to proteolytic digestion with proteinase K, PrP^Sc^ is not detectable in lymphoid tissue by immunohistochemical examination (IHC) and relatively absent in the brainstem. Different biochemical profiles are also identified on Western blot examination of brain (Benestad et al., 2008). Scrapie is a World Organization for Animal Health (OIE) listed disease, although management of the animal health risks associated with the scrapie agent in the OIE’s Terrestrial Animal Health Code (OIE, 2018) only applies to classical scrapie, due to the known contagious nature of this disease type. In Europe, rules for the prevention, control, and eradication of classical scrapie are laid down in European Parliament and Council Regulation (EC) No 999/2001. In addition to the examination of reported clinical suspects, this requires active surveillance for TSEs by testing brain tissue from healthy slaughter animals or animals that died or were killed on farm (fallen stock), where surveillance stream and minimum number of tested animals is dependent on the animal population in each member state. In the United Kingdom, a minimum of 500 fallen goats over 18 months of age need to be tested. Regulation (EC) No 999/2001 currently lists four rapid TSE tests that can be used for brain examination. In case of a positive result, the sample requires examination by confirmatory tests, such as Western immunoblot (WB) or immunohistochemistry (European Parliament Council of the European Union, 2001).

If classical scrapie is confirmed in a holding, there are different options for disease eradication: complete herd cull, cull of susceptible animals only (in sheep), or no cull provided that animals, at the end of their productive lives, are slaughtered in the country of origin and those over 18 months of age are tested for scrapie.

Susceptibility to scrapie is influenced by polymorphisms in the prion protein gene (*PRNP*), principally at codons 136, 154, and 171, and selection for resistant genotypes has led to a great reduction in classical scrapie cases in sheep within the European Union (EFSA, 2014). Recent research has shown that there are also polymorphisms associated with lower risk toward classical scrapie in the caprine *PRNP*: at codon 142 [methionine (M) instead of isoleucine (I)], at codon 146 [serine (S) or aspartate (D) instead of asparagine (N)], at codon 154 [histidine (H) instead of arginine (R)], at codon 211 [glutamine (Q) instead of R] and 222 [lysine (K) instead of Q] (Barillet et al., 2009). Based on current scientific evidence, the K_222_, D_146_, and S_146_ alleles have been shown to confer genetic resistance to classical scrapie strains known to occur in the EU goat population [EFSA Panel on Biological Hazards (BIOHAZ) et al., 2017] although genotype-based scrapie eradication is currently not an approved option for goats in the EU regulation.

There are only a few reports on classical scrapie outbreaks in large goat herds, where goats were subject to clinical examinations (González et al., 2009) and postmortem examinations, which included assessment of PrP^Sc^ distribution in lymphoid tissue (González et al., 2009, 2010; Corbière et al., 2013a, b; Ortiz-Pelaez et al., 2015). These demonstrated that up to 50% of goats might be infected, even though the brain examination was negative. It has also been shown that brain examination by immunohistochemistry may be superior to testing by rapid tests in goats with classical scrapie (González et al., 2009; Ortiz-Pelaez et al., 2015).

Following an outbreak of classical scrapie in a goat farm in Great Britain in 2012, the decision was made to cull the whole herd (more than 2000 goats) which required the testing of a minimum subset of 150 goats over 18 months of age, according to Regulation (EC) No 999/2001. The study reported here describes the outcome of further investigations of this subset.

## Materials and Methods

### Herd and Case History

The affected farm was a dairy goat herd with 2500 goats. It was established in 2007 by purchasing 600 female goats from one farm and five male goats from another farm. More goats were subsequently purchased from eight different farms to increase herd size, and female and male goats from other farms were added to the herd annually. According to the owner, all goats were purchased from herds that had been monitored for scrapie. The main breeds were Saanen, Toggenburg, and Alpine. Pregnant does gave birth indoors. Adult does were routinely vaccinated against clostridial disease (Heptavac Plus, MSD Animal Health, Milton Keynes, United Kingdom) and enzootic abortion (Cevac Chlamydia, Ceva Animal Health, Amersham, United Kingdom). The farm had a history of sheep occupancy, although there was no known history of scrapie in these sheep. Classical scrapie was first detected in a fallen stock goat in March 2012, which resulted in TSE monitoring of all slaughtered or dead animals over 18 months of age. The scrapie prevalence based on postmortem test examination of the brain from clinical suspects and fallen stock over a 4-year period (2012–2015) was 2.8% in 2012, 3.6% in 2013, 2.7% in 2014, and 2.4% in 2015 (mean 2.9%; 95% confidence interval: 2.06, 3.69).

In 2014, the farmer took part in the survey of the national goat population to determine the proportion of goats with a scrapie resistant K_222_ allele of the *PRNP* (Goldmann et al., 2016) but none of the 16 billies and 14 does used for breeding carried this allele.

The farmer reported the first clinical suspect in March 2015, which was confirmed positive. Eleven further cases were reported as clinical suspects up to January 2016, of which five tested positive for scrapie based on examination of the brain. One goat that tested negative in brain had lymphoid tissue tested retrospectively, and presented with PrP^Sc^ by immunohistochemistry in medial retropharyngeal lymph node (LN), mesenteric LN, spleen, distal ileum, and recto-anal mucosa-associated lymphoid tissue (RAMALT).

In February 2016, after it was decided to cull the herd, 151 female goats were transported to APHA Weybridge for clinical examination and more extensive investigation by postmortem tests. Goats were to some degree selected randomly from 920 lactating and 25 non-lactating goats (the farmer was asked to pick the goats randomly), but the group included any goats with possible clinical signs of scrapie, and all the goats selected were at least 24 months old (minimum age of previous confirmed scrapie cases) and born on the farm, even though at least five of 56 previously confirmed scrapie cases were born on a different farm.

Herd cull was carried out under Regulation (EC) No. 999/2001 and the relevant national Transmissible Spongiform Encephalopathies (England) Regulations to eradicate scrapie and no licensed procedures were undertaken in animals that would have required ethical approval. However, the same standard for animal care and housing that is generally applied to animals used for scientific procedures under the Animal (Scientific Procedures) Act 1986 was applied.

### Clinical Assessment

Although goats were assessed at least twice daily by animal technicians during normal husbandry procedures, specific clinical examinations for signs of scrapie were only done once prior to cull and provided the clinical data for analysis. Goats were group-housed and examined in their pen for between 1 and 51 days after arrival (median 22 days) and up to 50 days (median 5 days) prior to cull. Cull had to be scheduled over 63 days due to availability of staff and postmortem room, and goats with clinical disease (not necessarily scrapie) or those that were heavily lactating were culled first. All goats were female with a median age of 36 months (range: 24–72 months) based on the farmer’s records. There were 102 (68%) lactating goats, which were milked daily and dried off depending on cull date and milk volume generated. A short examination protocol was used to assess body condition, posture and movement, behavior, pruritus, and vision (Konold and Phelan, 2014), which differed from the one used previously (Konold et al., 2010) in that it also included blindfolding to assess vestibular system function. Animal handling was limited to the assessment of the menace response, scratch test, body condition, and response to blindfolding, and the animal was visually inspected in the pen to assess behavior, locomotion, tremor, hair or skin changes, and response to a hand clap. Based on the observed signs, an animal was regarded as “showing no evidence of scrapie”, “inconclusive with regards to scrapie”, or “clinical suspect” prior to cull. To be inconclusive, the animal had to display one of the signs: repeatable response to scratching (positive scratch test), tremor, abnormal behavior, circling, collapsing episodes, ataxia/dysmetria, or a uni- or bilateral absent menace response, whereas a suspect had to clearly and consistently display more than one of these signs (Konold and Phelan, 2014).

The clinical diagnosis was compared with the postmortem diagnosis (see below) to establish diagnostic sensitivity (percentage of animals with scrapie that have disease suspicion based on clinical signs) and specificity (percentage of animals without scrapie that do not have suspicious signs of scrapie).

### Postmortem Examination

Goats were euthanized by intravenous barbiturate overdose (10 ml Somulose, Dechra, Shrewsbury, United Kingdom) and exsanguination. The following tissues were collected: brain, lateral and medial retropharyngeal LN, submandibular LN, palatine tonsil, pre-scapular LN, pre-femoral LN, mesenteric LN, spleen, distal ileum, and rectal tissue containing RAMALT. The brain was cut sagittally, the right side fixed with 10% formol saline and the left side frozen. Lymphoid tissue was fixed in 10% buffered formalin. The right side of the obex was submitted to an approved TSE testing site (Eurofins Forensic Services, Risley, United Kingdom) for rapid testing (TeSeE ELISA, Bio-Rad Laboratories, Watford, United Kingdom) to detect the proteinase-resistant prion protein PrP^res^. Tissue processing for IHC with monoclonal antibody R145 (APHA Weybridge, New Haw, United Kingdom) was as described previously for brain (Dustan et al., 2008). R145 is a rat monoclonal antibody, which recognizes the epitope 231-RESQA-235 of the bovine prion protein and was applied to sections for 60 min at a dilution of 1:150. Brain sections for IHC included the medulla oblongata at the level of the obex (all cases), rostral medulla at the level of the cerebellar peduncles, cerebellum, thalamus, and cerebrum at the level of frontal cortex (all cases with no detectable PrP^Sc^ in the obex). All obex samples that were tested by rapid test were also tested by discriminatory WB using the methodology described previously (Simmons et al., 2016), with the exception of two goats where caudal medulla was tested instead due to lack of remaining obex. The WB is based on the Bio-Rad TeSeE Universal Western Blot using the manufacturer’s instructions. Two replicate gels were run with 18.75 mg tissue equivalent per well. Primary antibodies were Sha31 (Bio-Rad Laboratories, directed against the 148-YEDRYYRE-155 epitope), which was prepared according to the manufacturer’s kit instructions, and the N-terminal antibody P4 (R-Biopharm Rhône Ltd., Glasgow, United Kingdom, directed against the 93-WGQGGSH-99 epitope), which was used in a dilution 1:5000 of a stock solution of 1 mg/ml. Incubation times for the Sha31 and P4 antibodies were 30 and 60 min, respectively. Additional investigations were requested for clinical suspects with neurological signs in which scrapie was not confirmed, which included staining of brain sections with hematoxylin-eosin (Dustan et al., 2008) and gram-staining in cases with inflammatory changes (Stevens and Bancroft, 1982).

A 1 g piece of frontal cortex was submitted fresh from each goat to determine the full open reading frame of the caprine *PRNP* using the same equipment, reagents, primers, and protocol as published previously (Konold et al., 2010). For the purpose of this study, polymorphisms are reported for codons 142, 146, 154, 211, 222, and 240.

### Statistical Calculations

All calculations were carried out using Statistica (version 13.5, TIBCO, Dublin, Ireland).

To find retrospectively the best clinical markers for disease suspicion using confirmatory tests on brain as gold standard (see below), classification and regression tree analysis was used (Breiman et al., 1984). Categorical dependent variables were the TSE status based on brain examination (positive, negative), and factor codes were the clinical variables tremor (yes, no), mental status (normal, abnormal), behavior resting/approached/handled (normal, abnormal), menace response (normal, impaired, exaggerated), scratch test (negative, positive, inconclusive), body condition (good, poor), ataxia (no, yes), circling or loss of balance when blindfolded (yes, no), skin lesions (yes, no), skin lesion frequency (none, one, more than one), hair loss (yes, no), hair loss frequency (none, one, more than one), short hair (yes, no), and short hair frequency (none, one, more than one). Equivocal signs (e.g., inconsistent menace response, temporary circling when blindfolded or possibly ataxia) were still classified as abnormal.

To compare the age of goats between groups (scrapie-negative, scrapie-positive in lymphoid tissue only, scrapie-positive in brain), age distribution was assessed for normality in box and whisker plots and an appropriate test used (ANOVA or the non-parametric Kruskall–Wallis if data were not normally distributed).

To assess whether false negative results in the screening ELISA test were associated with higher optical density (OD) values than true negative results, OD values for each sample were divided by the cut-off specified for each test run to calculate an OD cut-off ratio (multiplied by 100). The ratios were grouped by *PRNP* genotype at codon 142 based on recent findings that *PRNP* polymorphisms may affect test sensitivity in goats (Simmons et al., 2020), and the data compared by non-parametric Mann–Whitney *U*-test. This test was also used to compare the ages of goats with a false negative and true negative ELISA result, grouped by genotype at codon 142.

To determine whether scrapie status was associated with particular genotypes, the proportion of scrapie-positive and negative animals carrying each polymorphism was compared by Fisher’s exact test; *P* < 0.05 was indicative of an association of genotype with scrapie status.

## Results

All data can be found as a supplementary file (Supplementary Table S1). Animal data, test results, and clinical status of these scrapie cases are displayed in Table 1.

**TABLE 1:** Scrapie case data.

**Goat ID**	**Breed**	**Age [m]**	***PRNP* Genotype**	**ELISA (obex)**	**IHC obex**	**WB (obex or caudal medulla)**	**IHC (lymphoid tissue)**	**Clinical status**
2113	TO	60	II_142_ PP_240_	**P**	**P**	**P**	**P**	**Suspect^I^**
2135	SA	36	II_142_ PP_240_	**P**	**P**	**P**	**P**	**Suspect**
2073	TO	60	II_142_ SP_240_	**P**	**P**	P**	**P**	Inconclusive
2146	SA	24	II_142_ PP_240_	N	P*	N**	**P**	Inconclusive
2165	AL	24	II_142_ SP_240_	N	P*	Inconclusive	**P**	No evidence
2176	TO	24	II_142_ SS_240_	N	**P**	**P**	**P**	No evidence
2078	AL	72	II_142_ PP_240_	N	**P**	**P**	**P**	No evidence
2117	SA	24	IM_142_ PP_240_	N	N	**P**	**P**	No evidence
2191	TO	60	II_142_ SP_240_	N	N	N	**P**	**Suspect**
2169	SA	60	IM_142_ PP_240_	N	N	N	**P**	**Suspect^II^**
2179	AL	24	II_142_ SP_240_	N	N	N	**P**	Inconclusive
2112	SA	60	II_142_ PP_240_	N	N	N	**P**	Inconclusive
2166	TO	24	IM_142_ SP_240_	N	N	N	**P**	No evidence
2201	TO	60	IM_142_ PP_240_	N	N	N	**P**	No evidence
2126	SA	60	II_142_ PP_240_	N	N	N	**P**	No evidence
2182	SA	24	II_142_ PP_240_	N	N	N	**P**	No evidence
2143	TO	60	II_142_ SP_240_	N	N	N	**P**	No evidence
2155	TO	24	II_142_ SP_240_	N	N	N	**P**	No evidence

AL = Alpine, SA = Saanen, TO = Toggenburg. All goats were GG_127_, RR_211_, and QQ_222_. *Minimal immunolabeling; **caudal medulla was tested; P = positive, N = negative; one WB result was classified as inconclusive because of an extremely weak band at approximately 27 kDa (the PrP^res^ region), ^I^video clip “classical scrapie in goats” of this animal available to view at https://www.youtube.com/watch?v=Kkhak9yJ3H0 (Konold and Vallino Costassa, 2018); ^II^see Supplementary Video File S1.

Following the clinical examination, 12 goats (8%) were classified as clinical suspects, 32 (21%) were inconclusive with regard to scrapie, and 107 (71%) showed no evidence of scrapie.

The pathological examination identified 18 goats (11.9%) with PrP^Sc^ indicative of infection with the scrapie agent.

Three goats (2.0%) tested positive on brain examination by the rapid test, confirmed by IHC and WB. Five goats (3.3%) were rapid test-negative but positive on the confirmatory tests.

**FIGURE 1:**
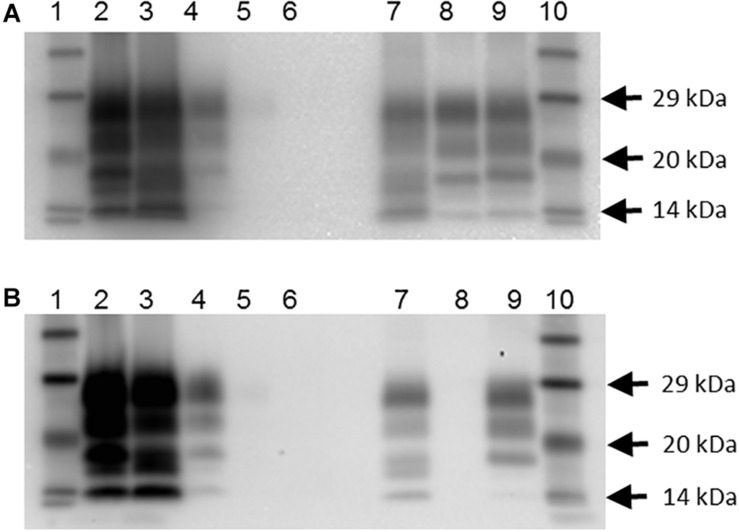
Western blot profile of digested brain samples from selected goats using two monoclonal antibodies. **(A)** Sha31 antibody. **(B)** P4 antibody. Lanes 1 and 10: molecular mass marker; lane 2: goat 2135 (clinical suspect, positive on brain by IHC and ELISA); lane 3: goat 2113, clinical suspect, positive on brain by IHC and ELISA); lane 4: goat 2078 (no clinical signs of scrapie, positive on brain by IHC, negative by ELISA); lane 5: goat 2117 (no clinical signs of scrapie, negative on brain by IHC and ELISA, positive on lymphoid tissue by IHC; scrapie profile difficult to discern with the picture contrast used); lane 6: goat 2102 (clinical suspect, negative on brain by ELISA and on all tissues by IHC); lane 7: caprine classical scrapie control (RSCRAP 17/00006, II_142_QQ_222_); lane 8: bovine classical BSE control (RBSE 98/00291); lane 9: ovine classical scrapie control (PG1903/97).

**FIGURE 2:**
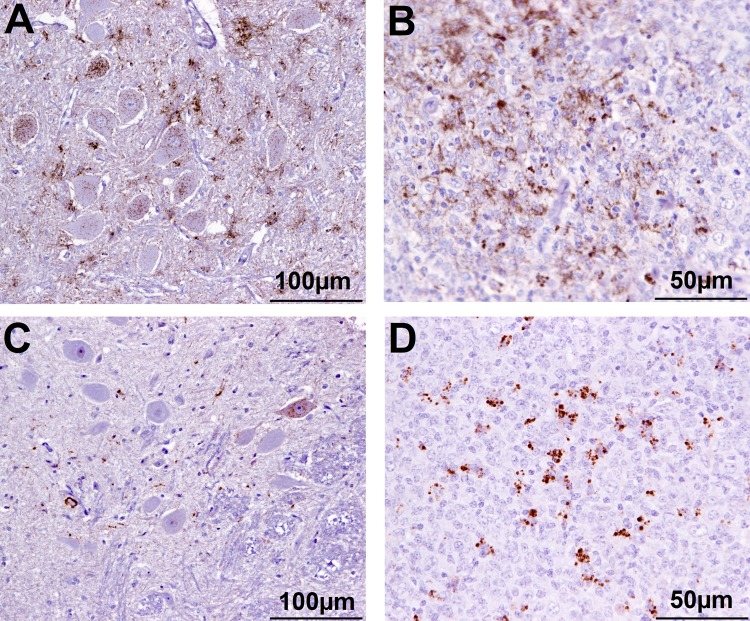
Immunohistochemical examination of brain and lymphoid tissue from selected goats. Obex **(A)** and medial retropharyngeal LN **(B)** from clinical suspect goat 2135, positive on brain by ELISA and WB; obex **(C)** from goat 2146 with inconclusive signs with regard to scrapie, negative on brain by ELISA and WB and pre-scapular LN **(D)** from clinical suspect goat 2169, negative by all tests on brain, PrP^Sc^ detected in pre-scapular LN only. Note the comparatively sparser PrP^Sc^ accumulation in the obex in **C** (immunolabeling visible in only one neuron) compared to **A**. Immmunolabeling is restricted to tingible body macrophages in the pre-scapular LN in **D** whereas macrophages and follicular dendritic cells are both immunolabeled in **B**.

In addition, there were 10 goats (6.6%), which had PrP^Sc^ in lymphoid tissue, but brain examination was negative. The WB profile (see Figure 1 for examples) or presence of PrP^Sc^ in lymphoid tissue (see Figure 2 for examples) was suggestive of classical scrapie in all cases.

Two goats (2153—scrapie-negative and 2179—scrapie-positive in lymphoid tissue only) had encephalitic lesions in the brain suggestive of listeriosis, and gram-staining identified gram-positive rods in 2153. Goat 2153, which presented with multifocal severe suppurative encephalitis, developed neurological signs 7 days after being examined for scrapie, which included inability to stand unaided, head tilt and turn to the right and vertical nystagmus suggestive of a vestibular disease, and was euthanized. It had been clinical unremarkable with no evidence of scrapie at the time of the examination for scrapie. Goat 2179, which presented with moderate focal necrotizing, granulomatous encephalitis, was euthanized 5 days after the examination and considered inconclusive with regard to scrapie because of the display of a head tremor and temporary anti-clockwise circling when blindfolded. It presented with PrP^Sc^ in lymphoid tissue. By contrast, a goat (2102) that displayed a head tremor and ataxia with hind limb weakness, which was considered a scrapie suspect, had neither scrapie confirmed nor any other noticeable lesions in the brain.

All cases that were positive by examination of the brain also had PrP^Sc^ in lymphoid tissue. A total of 144 goats (95%) had the whole range of lymphoid tissue suitable for examination. The tissue that presented with PrP^Sc^ most frequently was the medial retropharyngeal LN, the least frequent the spleen (see Table 2). PrP^Sc^ was detected in all 10 lymphoid tissues in the three cases with a positive brain ELISA result and in one case with a negative ELISA but positive confirmatory brain test result. It was more variable in the other scrapie cases and one goat (2169) had detectable PrP^Sc^ only in the pre-scapular LN.

**TABLE 2:** PrP^Sc^ accumulation in ten peripheral issues in the 18 scrapie-positive goats.

**Tissue (N examined)**	**PrP^Sc^ positive (% of scrapie-affected goats)**
Medial retropharyngeal LN (151)	17 (94.4%)
Palatine tonsil (151)	16 (88.9%)
Submandibular LN (149)	13 (72.2%)
Mesenteric LN (151)	13 (72.2%)
Pre-scapular LN (151)	13 (72.2%)
Distal ileum (151)	12 (66.7%)
Pre-femoral LN (151)	11 (61.1%)
Lateral retropharyngeal LN (148)	9 (50.0%)
RAMALT (149)	9 (50.0%)
Spleen (151)	7 (38.9%)

The mean age of animals positive for scrapie in brain (*N* = 8, mean 41 months, range 24–72 months), positive in lymphoid tissue only (*N* = 10, mean 46 months, range 24–60 months), and scrapie-negative animals (*N* = 133, mean 39 months, range 24–72 months) was not significantly different (*P* = 0.67, Kruskall–Wallis test on not normally distributed ages).

Sensitivity and specificity of the clinical examination were 25 and 93%, respectively, using brain examination as the gold standard and only clinical suspects as scrapie-affected. Inclusion of clinically inconclusive cases as also scrapie-affected would double sensitivity but reduce specificity to 72% with the brain examination as gold standard.

**FIGURE 3:**
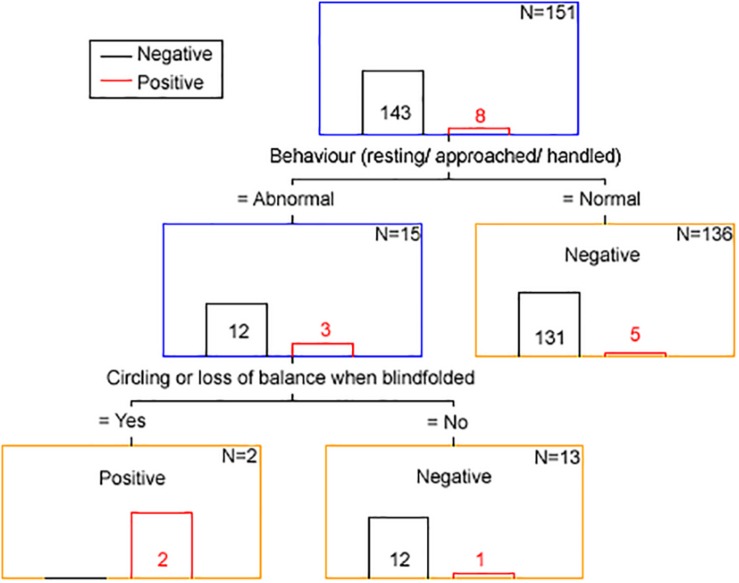
Clinical discriminator for scrapie status based on classification and regression tree analysis. The number in or above each bar is the number of animals with a positive (red font and bar) and negative (black font and bar) scrapie status based on examination of brain tissue by all confirmatory tests. *N* is the number of animals remaining in each node (blue box = intermediary node, yellow box = final node). The term “Negative” of “Positive” in each node corresponds to the clinical scrapie status.

The results from the classification and regression tree analysis are displayed in Figure 3.

Using the clinical discriminators “behavior resting/approached/handled” and “circling or loss of balance when blindfolded” 143 goats were correctly identified as brain-negative (100% specificity), two were correctly identified as brain-positive and six were falsely identified as brain-negative (25% sensitivity).

A list of the clinical signs displayed by scrapie-positive and negative goats is shown in Table 3.

**TABLE 3:** Individual signs in 151 goats assessed by the short protocol.

	**Scrapie-positive (brain) *N* = 8**	**Scrapie-positive (LRS only) *N* = 10**	**Scrapie-negative *N* = 133**
Abnormal behavior	3 (37.5%)	2 (20.0%)	10 (7.5%)
Circling when blindfolded	2 (25.0%)	3 (30.0%)	3 (2.3%)
Menace response			
Impaired	2 (25.0%)	0	26 (19.5%)
Exaggerated	1 (12.5%)	3 (30.0%)	16 (12.0%)
Tremor	1 (12.5%)	4 (40.0%)	14 (10.5%)
Abnormal mental status	1 (12.5%)	0	2 (1.5%)
Disequilibrium when blindfolded	1 (12.5%)	0	0
Ataxia or dysmetria	1 (12.5%)	1 (10.0%)	2 (1.5%)
Poor body condition (BCS ≤ 2)	0	3 (30.0%)	8 (6.0%)
Scratch test			
Positive or inconsistent	0	0	8 (6.0%)
Inconclusive	0	0	1 (0.8%)
Hair loss			
Poll	4 (50.0%)	5 (50.0%)	68 (51.1%)
Nose	1 (12.5%)	0	10 (7.5%)
Neck	0	1 (10.0%)	2 (1.5%)
Side abdomen/chest	0	1 (10.0%)	1 (0.8%)
Rump	0	1 (10.0%)	1 (0.8%)
Tail base	0	1 (10.0%)	0
Eyelid	0	0	7 (5.3%)
Shoulder	0	0	2 (1.5%)
Back	0	0	1 (0.8%)
Hair short			
Shoulder	1 (12.5%)	0	4 (3.0%)
Neck	0	2 (20.0%)	11 (8.3%)
Rump	0	1 (10.0%)	9 (6.8%)
Poll	0	1 (10.0%)	5 (3.8%)
Side abdomen/chest	0	1 (10.0%)	1 (0.8%)
Tail base	0	0	16 (12.0%)
Eyelid	0	0	1 (0.8%)
Back	0	0	1 (0.8%)

A significantly higher proportion of lymphoid tissue only-positive goats displayed tremor, and a significantly higher proportion of brain-positive goats displayed abnormal behavior, compared to the scrapie-negative group (two-tailed Fisher’s exact test, *P* = 0.046 after applying Bonferroni’s correction for multiple comparisons).

Table 4 lists the number and percentage of scrapie-positive and negative goats with polymorphisms associated with resistance to classical scrapie. A significantly higher proportion of homozygous I_142_ goats compared to heterozygous or homozygous M_142_ goats were affected by scrapie [two-tailed Fisher’s exact test, *P* = 0.008 (II_142_ versus IM_142_) and 0.017 (II_142_ versus MM_142_) after applying Bonferroni’s correction for multiple comparisons]. None of the goats carried an H_154_ allele.

**TABLE 4:** Number and frequency of goats with selected allele variants.

	**Number of goats with allele variants (proportion)**	
**Polymorphism at codon**	**Scrapie-positive (18)**	**Scrapie-negative (133)**
II_142_	14 (78%)	44 (33%)
IM_142_	4 (22%)	67 (50%)
MM_142_	0	22 (17%)
IM_142_ PP_240_	2 (4%)	53 (96%)
IM_142_ SP_240_	1 (6%)	15 (94%)
NN_146_	18 (100%)	131 (98%)
NS_146_	0	2 (2%)
RR_211_	18 (100%)	128 (96%)
RQ_211_	0	5 (4%)
QQ_222_	18 (100%)	130 (98%)
QK_222_	0	3 (2%)

When the OD value ratios were compared between four rapid test-negative but confirmatory test positive and 39 rapid and confirmatory test negative II_142_ goats, there was no significant difference in the OD ratios: median 8.4% (range: 8.1–12.6%) and median 9.4% (range: 3.0–18.7%), respectively; *P* = 0.95. The single IM_142_ goat with a false negative ELISA result had a ratio of 9.1%, which was close to the median of 64 goats with a true negative result (median: 9.4%, range: 5.5–29.9%). All these goats were RR_211_, QQ_222_ and PP_240_, SP_240_ or SS_240_. The median age of the four II_142_ ELISA-false negative goats was 24 months (range 24–72 months), which was not significantly different to the 39 true negative goats (median 36, range: 24–72 months) with the same genotype (*P* = 0.41). Similarly, the single ELISA-false-negative IM_142_ goat was 24 months of age, which was close to the median of the 64 true-negative IM_142_ goats (median 36, range 24–72 months).

## Discussion

Continued monitoring by postmortem tests and whole herd cull are currently the only options in case of a classical scrapie outbreak in goats, although they are unlikely to succeed in the eradication of classical scrapie in a country (EFSA, 2014). Both options are costly (compensation for culled goats *versus* costs for continuing monitoring for scrapie of fallen stock and healthy slaughter goats over 18 months) and both have considerable emotional impact on the farmer (herd cull versus continuous restrictions and animal welfare implications due to scrapie). In our case, whole herd cull was selected, which provided an opportunity for more information about classical scrapie in goats, complimentary to previous studies of a different goat herd in Great Britain (González et al., 2009; Konold et al., 2010), to compare the test sensitivity of clinical examinations and screening TSE postmortem tests with confirmatory tests and assess distribution of PrP^Sc^ in various tissues.

Based on the scrapie prevalence in this farm in the previous 4 years, which was below 4% and had declined over the previous 3 years, it was unexpected to find 5.3% of goats to be scrapie-affected based on brain examination alone. The selection of goats for transport was not completely random, and bias may have occurred because clinical suspects were included. Thus, the percentage of confirmed scrapie cases within the 151 goats may not reflect the true prevalence in the whole herd. However, this bias effect was likely to be small because of the high proportion of clinical suspects previously in this herd that were not confirmed pathologically.

Most striking was the finding that the ELISA, a postmortem screening test approved for TSE testing by the European Union, considerably underestimated the number of scrapie cases, with only three cases (2%) being positive whereas a further five cases were diagnosed by the confirmatory tests, IHC or WB, when testing the same brain region (obex). The 2% detection rate is in agreement with the estimated herd prevalence in previous years, which was also based on the ELISA as an initial screening test, but underestimates the real scrapie prevalence in this herd. Previous studies have demonstrated that caprine scrapie cases may be missed by reliance on the screening test alone (González et al., 2009; Ortiz-Pelaez et al., 2015) but misdiagnosis of more than 50% of cases in our study was unexpected, and it contradicts results from another study where ELISA and IHC performed similarly in the detection of scrapie in French goats (Corbière et al., 2013a). A recent study in goats demonstrated that rapid screening tests performed well in clinically affected animals but were less sensitive in the pre-clinical phase, which was dependent on *PRNP* polymorphisms. In particular, the presence of the M_142_ allele appeared to compromise to some extent the sensitivity of the Bio-Rad screening tests (Simmons et al., 2020). In the present study, however, four were II_142_ and only one was M_142_ heterozygous. There was no evidence that the ELISA-false negative cases had a higher OD value than the true negative cases, and it also did not seem to be influenced by breed and age. It confirmed disease in clinical suspects when PrP^Sc^ accumulation in the brain appears to be moderate to high as judged by IHC (Konold et al., 2010; Niedermeyer et al., 2016) but its reliability appears to be poor in asymptomatic cases, even goats that do not carry an M_142_ allele. Asymptomatic animals are likely to represent the majority of goats targeted by this screening test as part of the active scrapie surveillance. Of the two confirmatory tests used, WB and IHC, both performed equally well, with only one sample each classified as positive by one but not the other test. The WB-negative but IHC-positive sample was caudal medulla because obex was not available for WB testing in this animal, and testing of tissue caudal to the target area may explain a negative test result in the Western blot.

A further 10 goats presented with PrP^Sc^ in lymphoid tissue only, indicating infection with the scrapie agent and suggestive of an earlier stage of disease. This was expected from previous studies in goats where lymphoid tissue in addition to brain was tested (González et al., 2009; Corbière et al., 2013b; Ortiz-Pelaez et al., 2015; Niedermeyer et al., 2016; Georgiadou et al., 2017). Also in agreement with previous studies where at least five different lymphoid tissues were tested (González et al., 2009; Niedermeyer et al., 2016), the medial retropharyngeal LN was the tissue that most consistently presented with PrP^Sc^ by IHC, followed by palatine tonsil but there was no consistent hierarchy at an individual animal level. RAMALT was positive in 50% of the cases, which is similar to the 42% reported in an earlier study (González et al., 2009), but considerably lower compared to sheep with more than 85% (González et al., 2006), and, if used as *ante mortem* test, sensitivity may even be lower because of the considerably smaller amount of tissue that can be collected in a live animal.

One animal (2169) had detectable PrP^Sc^ only in the pre-scapular (superficial cervical) LN, which has previously been reported for a different scrapie-affected farm in the United Kingdom (González et al., 2010). As observed in this previous study for cases with limited lymphoid tissue involvement, immunolabeling was restricted to tingible body macrophages in this LN. It may indicate an earlier stage of infection as demonstrated for sheep with classical scrapie (van Keulen et al., 2002). Why only this LN was affected is not known; the same phenomenon was not observed in two other scrapie-affected goats with the same *PRNP* genotype. This may represent a different pathogenesis, for example, iatrogenic infection by subcutaneous injection with a contaminated needle in the neck region, of which afferent lymph vessels drain in this LN (Tanudimadja, 1973). It may also be caused by a different scrapie strain or some genetic or animal factors that led to limited PrP^Sc^ accumulation in peripheral LNs. This LN is generally not tested in scrapie studies so it is not known how often it occurs in scrapie outbreaks. Additional, deeper section examined from this LN by IHC were negative, which suggested that PrP^Sc^ accumulation was limited, possibly because of a very early stage of infection, and could easily be missed. Surprisingly, this goat was a clinical scrapie suspect displaying tremor, hair loss, mild ataxia, poor body condition, and clockwise circling when blindfolded. Histologically, there was diffuse mild vacuolation of the white matter tracts in thalamus, cerebrum, and cerebellum, occasionally accompanied by astrocytes with mild swollen vesicular astrocytes, which was interpreted as artifactual change although could have also been the result of a toxic or metabolic insult to the brain, but neither liver not kidney was available for further investigation.

None of the goats in the affected herd presented with PrP^Sc^ in brain only, without detectable PrP^Sc^ in lymphoid tissue, whereas in a previously reported outbreak of scrapie in British goats this was the case in four of 72 scrapie cases (6%), of which all were homo- or heterozygous M_142_ (González et al., 2009). This may be explained by the lower number of scrapie cases having these *PRNP* polymorphisms in the present study (three versus 32 in the other outbreak) or the scrapie strains were different.

A significantly greater proportion of goats with scrapie was homozygous for I_142_. There is some evidence that the scrapie risk is reduced in goats homozygous for M_142_ but also in goats heterozygous for M_142_ and homozygous for P_240_ (Barillet et al., 2009; Corbière et al., 2013b). However, in the current study, there were actually two goats with the IM_142_ PP_240_ combination that had scrapie compared to one goat with the IM_142_ SP_142_ combination. Indeed, a review of the literature concluded that the M_142_ allele is only associated with incomplete resistance to classical scrapie [EFSA BIOHAZ Panel (EFSA Panel on Biological Hazards (BIOHAZ) et al., 2017)]. None of the goats that carried a single allele associated with increased resistance (Q_211_, K_222_, S_146_) were scrapie-positive, which was expected from previous studies [EFSA Panel on Biological Hazards (BIOHAZ) et al., 2017] but the number of goats with these polymorphisms in the selected herd subpopulation was unexpected. The billies used on this farm did not carry a K_222_ allele, and to our knowledge artificial insemination was not used, which suggests that this allele must have been present in the female population and would have only been detected by genotyping more goats if a scrapie eradication program by genotype selection was considered. Given that the most recent national survey in the United Kingdom detected a QK_222_ genotype frequency of only 0.6% in Saanen, Toggenburg, and their crossbreds (Goldmann et al., 2016), which were the breeds present on the farm, finding 2% out of 151 goats with this polymorphism was unexpected. Similarly, the frequency of the NS_146_ genotype was mostly limited to Boer goats and in dairy goats was estimated to be 0.2% or less (Goldmann et al., 2011, 2016), whereas 2% (two Toggenburg goats) had this polymorphism in the study reported here.

Sensitivity of the clinical examination was expectedly poor although better than in a previous study where sensitivity was 3.9% (only clinical suspects considered scrapie-affected) and 11.7% (including clinically inconclusive goats) using brain examination as gold standard (Konold et al., 2010). This may be due to the inclusion of blindfolding in the short examination protocol, which as the classification tree suggested, was an important clinical discriminator. However, by including response to blindfolding, specificity, which was 99.6% (clinical suspects only) and 88.5% (with clinically inconclusive included) in the previous study, decreased slightly. Classification tree analysis indicated that only two clinical signs, abnormal behavior (nervousness or dullness) and abnormal responses to blindfolding (loss of balance or circling), were important for a suspect clinical diagnosis of scrapie with similar sensitivity although more examinations of goats with and without scrapie are necessary to confirm this. In general, circling is a sign more often observed in sheep with atypical scrapie (Onnasch et al., 2004; Konold et al., 2006; Simmons et al., 2010) than sheep or goats with classical scrapie (Laven, 1990; Ulvund, 2006; Konold et al., 2010) but blindfolding, which may elicit circling, is rarely used as part of the examination protocol.

None of the goats that tested positive for scrapie on brain showed signs of pruritus. Hair loss suggestive of pruritus was found in scrapie-positive and negative goats and the scratch test was only positive in scrapie-negative goats, which implied that scrapie goats displayed the non-pruritic form of the disease (Konold et al., 2010). It is possible that some of the scrapie-negative goats suffered from other conditions leading to hair loss and pruritus, such as mite or lice infestation (Jackson, 1986), which we did not investigate. Hair loss may also have non-infectious causes, such as friction due to poorly designed feeding stations. Indeed, a study in the United Kingdom has identified skin lesions, including hair loss, and pruritus as one of the major welfare issues in commercial dairy goat farms (Anzuino et al., 2010). It was surprising that neurological signs such as ataxia, tremor, and loss of balance or circling were not observed significantly more frequently in goats with PrP^Sc^ in brain. This may be due to other conditions that may have been present in the brain-negative population. For example, tremor in large animals as single sign may have no obvious cause and can even be a sign in frightened animals (Mayhew, 2009). It is also possible that some scrapie-affected goats were in an earlier stage of clinical disease when clear neurological signs may be more subtle, or absent, as demonstrated in a study where scrapie goats were monitored over time (Konold et al., 2010).

Listeriosis was diagnosed in two goats. In one goat neurological signs developed after the clinical examination and the clinical findings of nystagmus and head tilt were consistent with a vestibular system dysfunction as seen in listeriosis (Braun et al., 2002), which is unlike scrapie. Ideally, all clinical examinations should have been carried out within a few days prior to cull as clinical signs may develop after the clinical examination. This was not always possible for logistical reasons, but 87% of examinations were carried out within 14 days prior to cull. The other goat with listeriosis was clinically inconclusive because of the display of a head tremor and inconsistent circling when blindfolded. It implied that a neurological disease was present and a more thorough neurological examination might have detected other abnormalities associated with listeriosis, such as facial asymmetry and reduced sensory perception (Braun et al., 2002), or more signs developed over the 5 days between examination and cull.

## Conclusion

The results of this study continue to highlight the limitations of the Bio-Rad ELISA as brain screening test to diagnose classical scrapie in goats, and other or additional tests should be considered. It is recommended to include testing of the medial retropharyngeal LN or palatine tonsil, which are also located at the head that is generally submitted for testing and are less prone to rapid autolysis, to increase the sensitivity of goat scrapie surveillance.

## Data Availability Statement

This manuscript contains previously unpublished data. The name of the repository and accession number(s) are not available.

## Ethics Statement

Ethical review and approval was not required for the animal study because herd cull was carried out under Regulation (EC) No. 999/2001 and the relevant national Transmissible Spongiform Encephalopathies (England) Regulations to eradicate scrapie and no licensed procedures were undertaken in animals that would have required ethical approval. However, the same standard for animal care and housing that is generally applied to animals used for scientific procedures under the Animal (Scientific Procedures) Act 1986 was applied. Written informed consent for participation was not obtained from the owners because all animals were culled to eradicate disease and the owner compensated as per national TSE legislation. A subset of these animals were transported live to APHA Weybridge for culling and formed the basis for this study.

## Author Contributions

TK examined the goats and analyzed the data, supported by LP, and drafted the manuscript. JS, TF, BV, and MS carried out the histopathologic and immunohistochemical examinations. MS also managed the project. JT was responsible for Western immunoblotting, LF for genotyping, and NC for interpretation of the rapid test result. BR and JC provided historical data. All authors read, contributed to, and approved the final manuscript.

## Conflict of Interest

NC is employed by the company Eurofins.

The remaining authors declare that the research was conducted in the absence of any commercial or financial relationships that could be construed as a potential conflict of interest.

## Supplementary Material

TABLE S1 List of animal data with clinical signs and postmortem test results for each individual goat.

VIDEO FILE S1 Goat with PrP^Sc^ in pre-scapular lymph node only. This goat displays a whole body tremor, has extensive hair loss with skin lesions, suspected hind limb ataxia, and circles anti-clockwise when blindfolded.
